# The clinical application of PIVKA‐II in hepatocellular carcinoma and chronic liver diseases: A multi‐center study in China

**DOI:** 10.1002/jcla.24013

**Published:** 2021-09-30

**Authors:** Jun Ji, Lijuan Liu, Feifei Jiang, Xue Wen, Yu Zhang, Shengcong Li, Jinli Lou, Ying Wang, Ning Liu, Qiuyan Guo, Yongmei Jia, Chunfang Gao

**Affiliations:** ^1^ Department of Laboratory Medicine Eastern Hepatobiliary Surgery Hospital Second Military Medical University Shanghai China; ^2^ Department of Laboratory Medicine Mengchao hepatobiliary Hospital of Fujian Medical University Fujian China; ^3^ Center for Clinical Laboratory Beijing Youan Hospital Capital Medical University Beijing China

**Keywords:** chronic liver diseases, diagnostic evaluation, hepatocellular carcinoma, multi‐center study, Protein induced by vitamin K absence or antagonist‐II

## Abstract

**Background:**

Due to the absence of specific symptoms and low survival rate, efficient biomarkers for hepatocellular carcinoma (HCC) diagnosis are urgently required. The purpose of this study was to evaluate the diagnostic performance of protein induced by vitamin K absence or antagonist‐II (PIVKA‐II) and to determine the optimal cutoff values for HBV infection‐related HCC.

**Methods:**

We conducted a cross‐sectional, multi‐center study in China to ascertain the cutoff value for HCC patients in the context of CHB‐ and HBV‐related cirrhosis. The receiver operating characteristic curve (ROC) and the area under the curve (AUC) were used to evaluate the diagnostic performance of PIVKA‐II.

**Results:**

This study enrolled 784 subjects and demonstrated that PIVKA‐II had a sensitivity of 84.08% and a specificity of 90.43% in diagnosis HCC from chronic liver diseases. PIVKA‐II at a cutoff of 37.5 mAU/mL yielded an AUC of 0.9737 (sensitivity 91.78% and specificity 96.30%) in discriminating HCC from chronic hepatitis B (CHB) patients. PIVKA‐II at a cutoff of 45 mAU/mL yielded an AUC of 0.9419 (sensitivity 77.46% and specificity 95.12%) in discriminating HCC‐ from HBV‐related cirrhosis patients. Furthermore, using a cutoff value of 40 mAU/mL for PIVKA‐II as an HCC marker, only 4.81% (15/312) was positive in chronic hepatitis and 12.80% (37/289) in cirrhosis patients, revealing the satisfactory specificity of PIVKA‐II in chronic liver disease of different etiologies.

**Conclusion:**

Our data indicated that PIVKA‐II had satisfactory diagnostic efficiencies and could be used as a screening or surveillance biomarker in HCC high‐risk population.

## Graphical abstract

Multi‐center study showed the satisfactory diagnostic performance of PIVKA‐II in hepatocellular carcinoma. PIVKA‐II has excellent specificity in distinguishing benign liver diseases. The distribution of PIVKA‐II among chronic liver diseases.

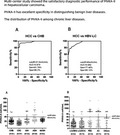

## 1 INTRODUCTION

In 2018, liver cancer ranked the sixth most common cancer and the fourth leading cause of cancer deaths in the worldwide. Every year, there are about 841,000 new cases and 782,000 deaths globally.^1^ Primary liver cancer can be divided into hepatocellular carcinoma (HCC), cholangiocarcinoma (ICC), and mixed type according to the pathological types, while HCC accounts for about 90% of all cases.^2^ As a high‐risk area of HCC, China contributes to more than half of the new incidence and mortality related to HCC annually, and the morbidity and mortality rates ranked the fourth and third in China, respectively.^3^ Due to the absence of specific symptoms and early diagnosis, 70%–80% of patients are often diagnosed at an advanced stage with a 5‐year survival rate less than 40% and a recurrence rate exceeding 60%.^4^ Thus, effective tools for HCC diagnosis and prognosis prediction are urgently required.

Although there are regional differences, the main risk factors for HCC include virus infection (hepatitis B virus [HBV], hepatitis C virus [HCV]), aflatoxin‐contaminated foodstuffs, alcoholism, obesity, and type 2 diabetes.^1^ HBV contributes the largest proportion to liver cancer mortality in East Asia, at 41%.^5^ The current HBV infection rate in China is 6.1%, accounting for almost 30% of the global number.^6^ Consequently, the key determinant of HCC in China is HBV infection. Simultaneously, liver fibrosis, developed from chronic hepatitis, is also a high‐risk factor with progressive liver function damage.^7^ According to guidelines for the prevention and treatment of chronic hepatitis B (version 2019) formulated by the Chinese Society of Infectious Diseases, the Chinese Society of Hepatology, and the Chinese Medical Association, it is recommended that populations with HBV infection or liver cirrhosis should be monitored HCC risk at least every 3–6 months.^8^

Alpha‐fetoprotein (AFP), synthesized by approximately half of HCC,^9^ is currently the most widely used serological marker for HCC detection. However, AFP concentration may remain normal in up to 40% of the patients, especially in the early stages.^10^ In contrast, an elevation of AFP concentration can be detected in embryonic carcinomas, gastric cancer, and lung cancer.^11^ Increased AFP concentrations can also be frequently noted in benign liver diseases, such as 15%‐45.6% in HBsAg‐positive patients^12^, ^13^ and 11%–47% in liver cirrhosis.^14^, ^15^ Since high risk of HCC is almost always presented in patients with chronic liver diseases, these characteristics limit its utility. Consequently, the AFP assay was recalled from the diagnostic criteria recommended by the “American Association for the Study of Liver Disease” (AASLD) and the “European Association for Study of the Liver” (EASL).^16^, ^17^

Protein induced by vitamin K absence or antagonist‐II (PIVKA‐II), also known as des‐gamma carboxyprothrombin (DCP), was first introduced as a novel HCC biomarker in 1984.^18^ With the development of research over the years, PIVKA‐II’s clinical significance in primary liver cancer diagnosis and management has been emphasized.^19^, ^20^ Furthermore, PIVKA‐II showed less tendency to be elevated in other chronic liver diseases, yielded a specificity around 90%‐95% when compared HCC with cirrhosis and chronic hepatitis.^21^, ^22^ Thus, monitoring HCC risk in CHB or cirrhosis patients by PIVKA‐II may be more specific than AFP.

Therefore, we conducted a cross‐sectional, multi‐center study to analyze the diagnostic performance of PIVKA‐II in China. In addition, we ascertained the cutoff value for HCC patients in the context of CHB‐ and HBV‐related cirrhosis.

## 2 MATERIALS AND METHODS

### 2.1 Study design and participants

This was a cross‐sectional, multi‐center study. The study protocol was approved by the institutional review board of Eastern Hepatobiliary Surgery Hospital Affiliated to The Second Military Medical University (Cohort A), Beijing Youan Hospital (Cohort B), and Mengchao Hepatobiliary Hospital of Fujian Medical University (Cohort C). All the samples were leftover samples, and informed consent process was not required for this type of samples according to institutional review board approval. A total of 784 subjects from the above three hospitals were enrolled in this study from October 2017 to September 2018. Thereinto, 183 cases were liver cancer samples, 312 cases were chronic hepatitis, and 289 cases were cirrhosis. All the clinical diagnostic criteria for each disease were followed the Chinese‐related guidelines.

All the study subjects met the following criteria: Chinese, age 18 and older, and regardless of gender. Exclusion criteria were as follows: acute hepatitis of any cause; concomitant HIV or other autoimmune diseases; patients received warfarin or vitamin K prior to testing; specimen contaminated or with sediment or flocculation; samples with insufficient volume; contrary to the diagnostic criteria of the Chinese‐related guidelines.

### 2.2 Specimen collection and testing methods

The serum samples were collected at the time of diagnosis without treatment and stored at −20°C or lower temperature immediately. PIVKA‐II concentrations were measured by the chemiluminescence enzyme immunoassay (CLEIA) (Lumipulse G1200, Fujirebio, Tokyo, Japan) and the chemiluminescent microparticle immunoassay (CMIA) (Architect i2000, Abbott Diagnosis, Abbott Park, America) following the manufacturer's protocol, respectively.

### 2.3 Performance evaluation

#### 2.3.1 Reproducibility

Four concentration sample pools including low concentration (≤40 mAU/mL), medium concentration (41–200 mAU/mL), high concentration (201–1000 mAU/mL), and super‐high concentration (1001–30,000 mAU/mL) were prepared and measured ten times on the first day to calculate their coefficient of variation (CV%) at three research centers respectively. The 4‐level samples were measured six times again on days 3 and 7 after storage at 4°C; and days 14 and 30 after storage at −20°C to satisfy the different storage needs of clinical samples.

#### 2.3.2 Dilution linearity

Fourteen high concentration (20,000–30,000 mAU/mL) sample pools were prepared to evaluate linearity of the assay. Double dilution methods were performed by diluting specimens (with the specimen diluent of Lumipulse or the Calibrator A (0 mAU/mL) of Architect) until 1:32 dilution. Linearity was evaluated as the coefficient of determination (*R* ^2^) between measured and expected values for the respective samples.^23^

### 2.4 Statistical analysis

All data were analyzed by GraphPad Prism 6 (GraphPad Software, La Jolla, CA) and Medcalc (MedCalc, Ostend, Belgium) software programs. The Mann‐Whitney U test was used for the differential analysis of PIVKA‐II concentration. Discrete variables were compared by contingency table analysis of the chi‐square test or Fisher's Exact Test. Correlation coefficients were calculated to observe the correlation between two assays. The receiver operating characteristic curve (ROC) and the area under the curve (AUC) were used to evaluate the diagnostic performance of PIVKA‐II. The optimal cutoff value was determined by Youden's index. Two‐sided settings were used for statistical analysis and *p *≤ 0.05 was considered as statistically significant.

## 3 RESULTS

### 3.1 Participates characteristics

**FIGURE 1:**
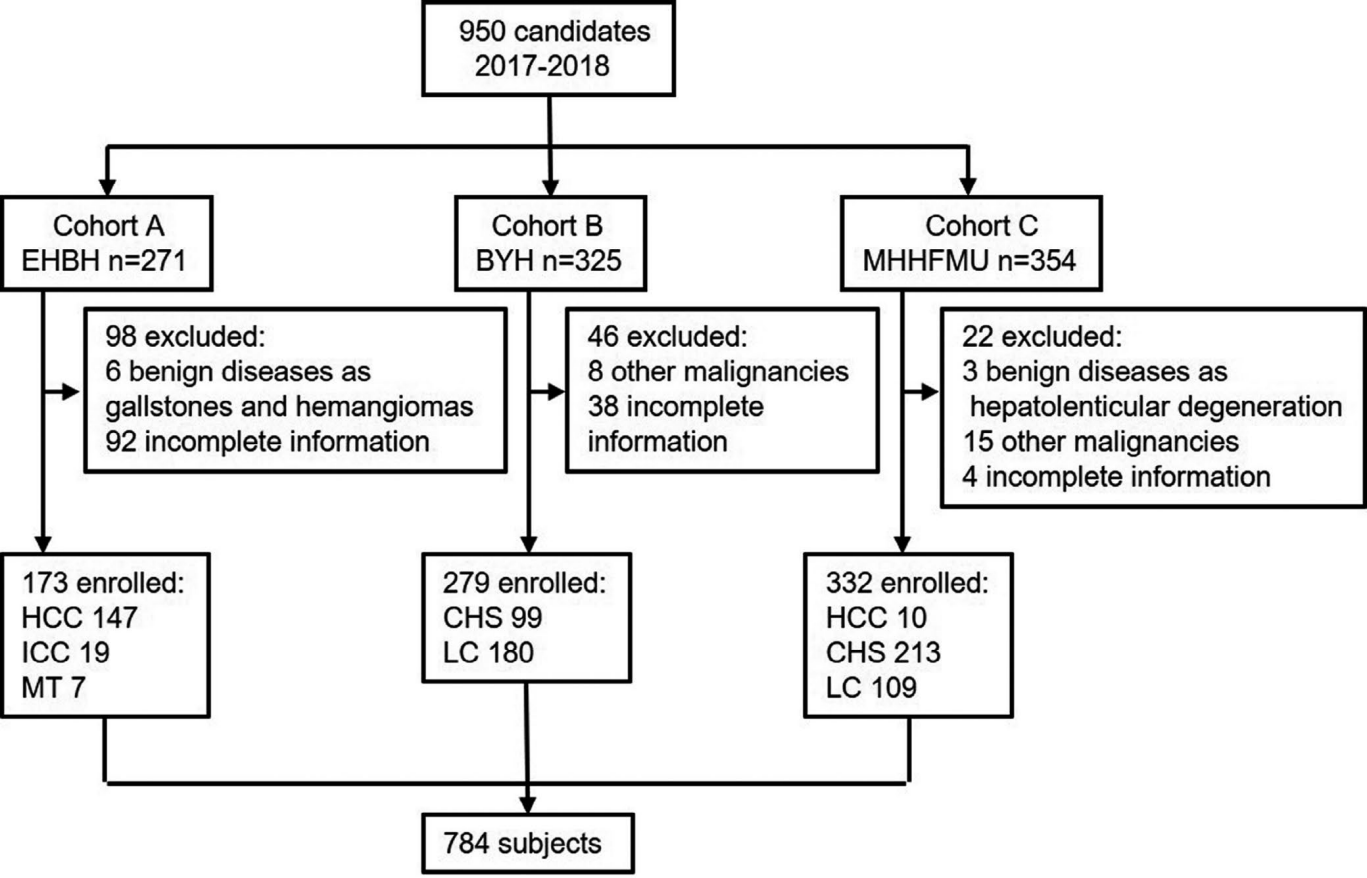
Study profile. Cohort A, Eastern Hepatobiliary Surgery Hospital; Cohort B, Beijing Youan Hospital; Cohort C, Mengchao Hepatobiliary Hospital of Fujian Medical University; HCC, hepatocellular carcinoma; ICC, intrahepatic cholangiocarcinoma; MT, liver metastasis; CH, chronic hepatitis; LC, liver cirrhosis

**FIGURE 2:**
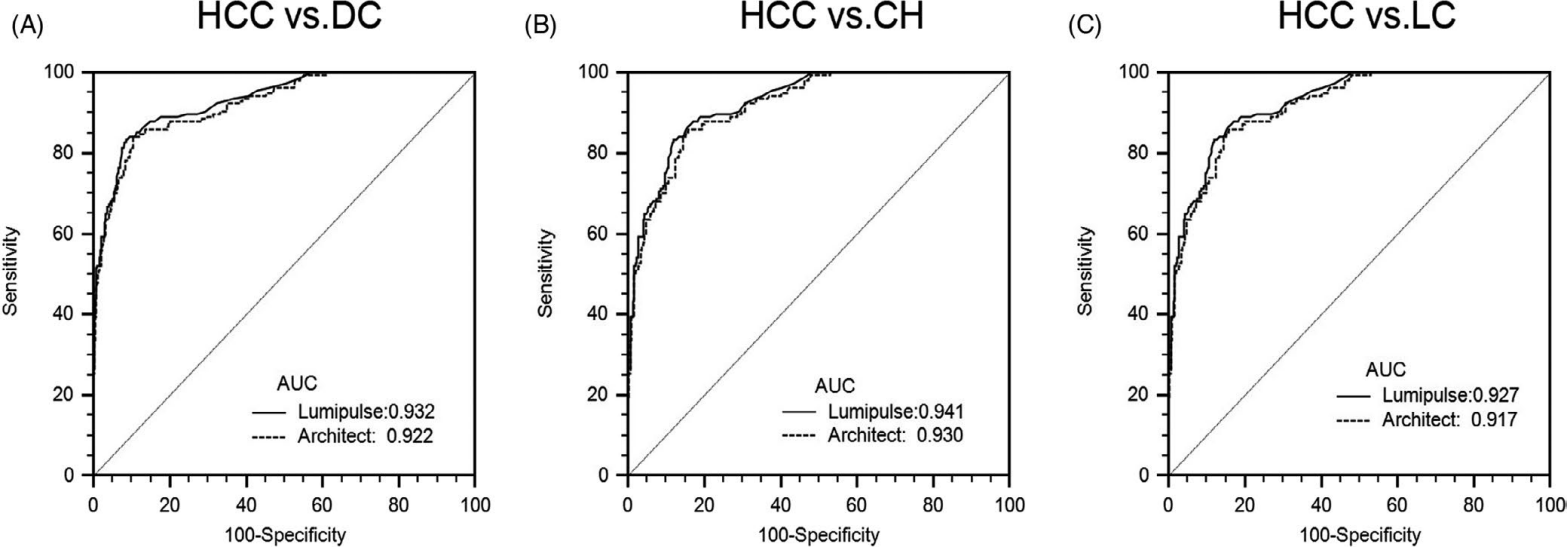
ROC analysis of two assays. Comparison of receiver‐operating characteristic curves (ROC) between two PIVKA‐II assays and their corresponding area under the curve (AUC) for (A) HCC vs. DC, (B) HCC vs. CH, (C) HCC vs. LC

**FIGURE 3:**
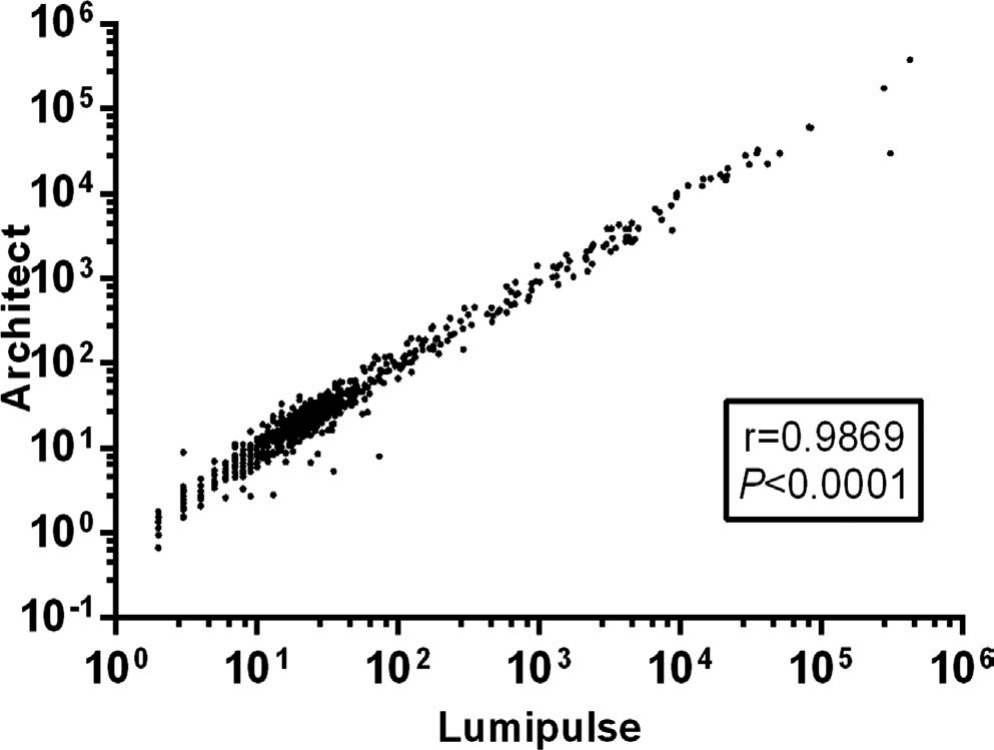
Correlation between two assay's measurement values. The correlation of two assays were evaluated by Pearson coefficient. All data had undergone logarithmic transformation

As shown in Figure 1, 2, 3, 950 individuals were included in the study, and 166 were excluded due to the incomplete information or other malignancies. Eventually, 784 participates were enrolled in the study. The basic characteristics of every participant were summarized in Table 1. Patients with liver cancer and cirrhosis were predominantly male (*p *< 0.0001), while the gender difference was not significant in patients with chronic hepatitis. Simultaneously, patients with liver cancer and cirrhosis were older than those with chronic hepatitis (*p *< 0.0001). According to the results of previous large‐scale studies,^24^ the concentrations of PIVKA‐II have no relationship with age or gender, and thus, we did not conduct further statistical processing on the samples. In HCC group, 91.7% cases had HBV infection, 3.18% cases had HCV infection, and 5.1% cases had other pathogeneses.

**TABLE 1:** Clinicopathologic features of participants

Variables	liver cancer group			Chronic Hepatitis	Liver Cirrhosis
	HCC	ICC	MT		
Sample size	157	19	7	312	289
Age (Mean ± SD)	54.0 ± 11.1	59.1 ± 9.5	51.7 ± 15.8	44.9 ± 14.1***	56.0 ± 10.5
Gender					
Male (%)	132(84.1)	13(68.4)	1(14.3)	172(55.13)***	186(64.4)***
Etiology					
HBV (%)	144(91.7)			81(25.96)	82(28.37)
HCV (%)	5(3.18)			87(27.88)	78(26.99)
HBV + HCV (%)					1(0.35)
Alcohol (%)				49(15.70)	70(24.22)
Non‐alcohol, non‐viral (%)				63(20.19)	
PBC (%)					39(13.49)
Others (%)	8(5.10)			32(10.26)	19(6.57)
BCLC					
A	3				
B	9				
C	130				
D	0				
Median (95% CI)	466 (194–841)	27 (24–36)***	23 (21–26)***	20 (19–22)***	17 (15–19.97)***

Note

BCLC staging of 142 HCC patients was collected. Compared with HCC group: *p < 0.05; **p < 0.01; ***p < 0.0001.

Abbreviations: HBV, hepatitis B virus; HCC, hepatocellular carcinoma; HCV, hepatitis C virus; ICC, intrahepatic cholangiocarcinoma; MT, liver metastasis; PBC, primary biliary cirrhosis.

The concentrations of PIVKA‐II were significantly higher in HCC group (median 466 mAU/mL, 95% CI 194–841) than other liver cancers (ICC: median 27 mAU/mL, 95% CI 24–36, *p *< 0.0001; MT: median 23 mAU/mL, 95% CI 21–26, *p *< 0.0001) and non‐HCC disease control (DC) groups (Chronic hepatitis [CHS]: median 20 mAU/mL, 95% CI 19–22, *p *< 0.0001; liver cirrhosis [LC] median 17 mAU/mL, 95% CI 15–19.97, *p *< 0.0001). Consistent with previous studies, our data revealed that the sensitivity of PIVKA‐II in ICC was 21.05%.^25^

### 3.2 Satisfactory diagnostic performance of PIVKA‐II

**FIGURE 4:**
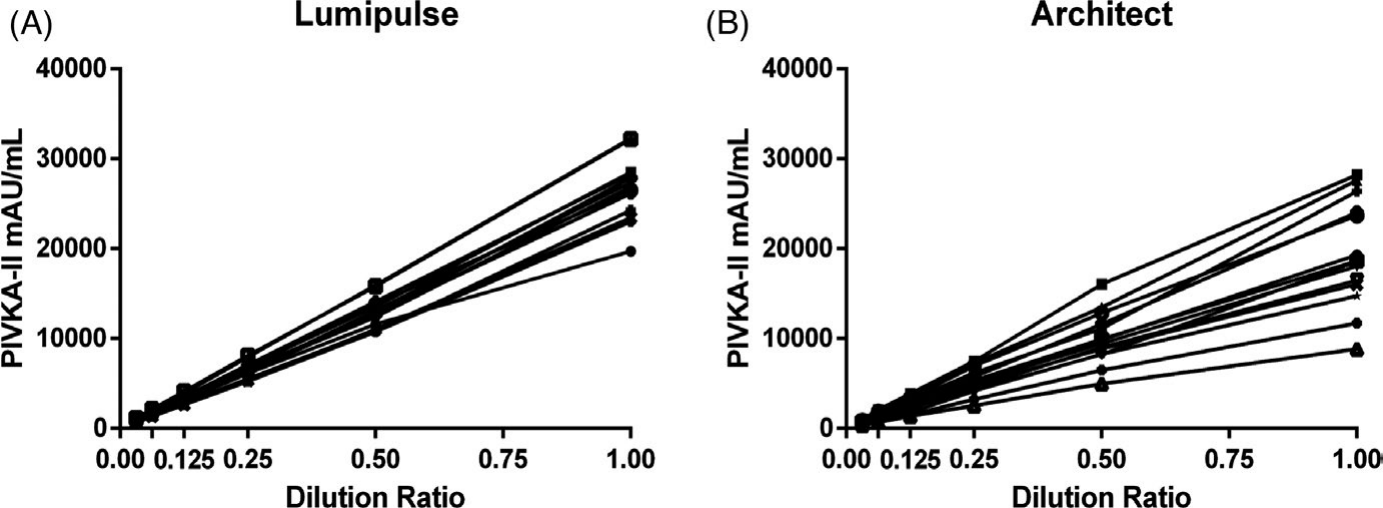
Dilution linearity. Linearity curves of dilution ratios vs actual values of PIVKA‐II measured by (A) Lumipulse, (B) Architect was shown. Each line represents one case of data

As shown in Figure 2 and Table 2, PIVKA‐II had a sensitivity of 84.08% and a specificity of 90.43% when distinguishing HCC from disease controls at the cutoff value of 40 mAU/mL; a sensitivity of 84.08% and a specificity of 94.55% when distinguishing HCC from patients with different etiology of chronic hepatitis; and a sensitivity of 84.08% and a specificity of 86.51% when distinguishing HCC from cirrhosis. As shown in Figure 3, the correlation coefficient of two PIVKA‐II assays was 0.9869. For the samples in various concentration ranges, the Lumipulse reagent showed the satisfactory reproducibility, regardless of stored in room temperature (RT) or different storage conditions. The CV values for all the concentrations and the conditions from three research centers were documented in Table 3. Figure 4 and Table 4 evaluated the dilution linearity of PIVKA‐II reagents. The coefficient of determination (*R* ^2^) between the measured values and the expected values of all the samples was 0.9917–0.9999 according to the Lumipulse's data.

**TABLE 2:** Performance of two PIVKA‐II assays in diagnosis of HCC from different controls

	Variables	AUC (95% CI)	Sen (%)	Spe (%)	Accuracy (%)	PPV (%)	NPV (%)
Lumipulse	HCC vs DC	0.9322 (0.912 to 0.949)	84.08	90.43	89.16	68.75	95.62
	HCC vs CH	0.9411 (0.916 to 0.961)	84.08	94.55	90.62	88.43	91.61
	HCC vs LC	0.927 (0.899 to 0.949)	84.08	86.51	85.65	77.20	90.91
Architect	HCC vs DC	0.922 (0.901 to 0.940)	82.17	89.63	88.14	66.49	95.26
	HCC vs CH	0.9297 (0.903 to 0.951)	82.17	93.59	89.77	86.58	91.25
	HCC vs LC	0.9168 (0.887 to 0.941)	82.17	85.47	84.31	75.44	89.82

Note

The diagnostic cutoff values of two PIVKA‐II assays were set at 40 mAU/mL.

Abbreviations: AUC, area under curve; CH, chronic hepatitis; DC, disease control; LC, liver cirrhosis; NPV, negative predictive value; PPV, positive predictive value; Sen, sensitivity; Spe, specificity.

**TABLE 3:** Reproducibility data of four concentration samples at different conditions measured by two PIVKA‐II assays

	Concentration	Institution	RT	4°C 3d	4°C 7d	−20°C 14d	−20°C 30d
Lumipulse	≤40 mAU/mL	Cohort A	6.03%	5.27%	5.87%	3.18%	4.02%
		Cohort B	2.65%	6.56%	2.99%	2.50%	3.16%
		Cohort C	3.76%	3.76%	6.88%	5.11%	2.43%
	41–200 mAU/mL	Cohort A	3.37%	2.59%	2.36%	3.13%	2.29%
		Cohort B	3.57%	3.86%	1.42%	2.76%	3.03%
		Cohort C	2.95%	2.66%	1.49%	2.91%	2.02%
	201–1000 mAU/mL	Cohort A	1.82%	2.01%	2.32%	2.24%	2.98%
		Cohort B	2.44%	3.30%	1.23%	2.48%	2.25%
		Cohort C	1.44%	1.51%	1.88%	2.35%	3.06%
	1001–30,000 mAU/mL	Cohort A	2.34%	5.03%	2.08%	1.86%	3.39%
		Cohort B	1.13%	3.95%	1.17%	2.64%	1.34%
		Cohort C	2.13%	1.96%	1.82%	2.08%	1.92%
Architect	≤40 mAU/mL	Cohort A	5.02%	5.96%	5.89%	3.25%	3.39%
		Cohort B	3.42%	2.68%	2.47%	3.82%	2.71%
		Cohort C	5.31%	9.85%	9.18%	5.69%	10.29%
	41–200 mAU/mL	Cohort A	4.54%	2.78%	2.96%	3.51%	3.08%
		Cohort B	3.44%	3.49%	4.56%	3.89%	2.66%
		Cohort C	6.34%	4.27%	5.61%	11.53%	4.05%
	201–1000 mAU/mL	Cohort A	2.11%	1.90%	2.95%	2.28%	4.58%
		Cohort B	3.16%	2.65%	5.05%	2.68%	2.50%
		Cohort C	4.27%	5.46%	4.80%	7.07%	1.81%
	1001–30,000 mAU/mL	Cohort A	3.75%	1.90%	4.67%	1.89%	2.03%
		Cohort B	3.37%	1.83%	7.40%	5.02%	1.86%
		Cohort C	4.94%	6.90%	3.58%	4.82%	2.86%

Abbreviations: CV, coefficient of variation; RT, room temperature.

**TABLE 4:** Linearity regression analysis of PIVKA‐II actual values measured by two PIVKA‐II assays vs expected values

	Variables	1	2	3	4	5	6	7	8	9	10	11	12	13	14
Lumipulse	*R* ^2^	0.9917	0.9992	0.9999	0.9991	0.9982	0.9997	0.9999	1	0.9996	0.9977	0.9988	0.9994	0.9964	0.999
	Slope	0.9928	0.9916	0.9986	0.9931	0.9886	0.9957	0.9529	0.9958	0.99	0.9975	0.9995	1.0041	0.9955	1.0004
	Intercept	757.96	−40.64	−37.43	−74.34	−82.31	268.07	108.39	81.51	102.79	−390.24	−255.64	−322.33	−394.14	−261.62
Architect	*R* ^2^	0.9998	0.9954	0.9996	0.9988	0.9963	0.9977	0.9984	0.9964	0.9979	0.9997	0.9971	0.9956	0.9931	0.996
	Slope	0.9987	1.0049	0.9939	0.9962	0.9878	0.991	0.9927	0.9906	0.9929	1.0026	1.003	1.0042	0.9835	0.9987
	Intercept	94.37	495.91	84.10	235.29	−158.29	573.79	365.48	266.18	368.50	−201.59	178.86	258.48	−292.45	339.20

We also analyzed the diagnostic performance of AFP. AFP had a sensitivity of 61.33% and a specificity of 91.15% when distinguishing HCC from disease controls at the cutoff value of 20 ng/mL (Table 5). Combined with PIVKA‐II, the sensitivity of AFP was increased to 89.33%, the AUC was increased from 0.817 (95% CI 0.773–0.862) to 0.859 (95% CI 0.825–0.894).

**TABLE 5:** Sensitivity and specificity of AFP and PIVKA‐II in the diagnosis of HCC

	AFP	PIVKA‐II	AFP + PIVKA‐II
Sen (%)	61.33	82.67	89.33
Spe (%)	91.15	90.00	82.50
AUC (95% CI)	0.817 (0.773–0.862)	0.928 (0.906–0.951)	0.859 (0.825–0.894)

Note

The diagnostic cutoff values of AFP and PIVKA‐II were 20 ng/mL and 40 mAU/mL. The results were collected from 150 HCC patients and 520 disease controls.

### 3.3 The distribution of PIVKA‐II among chronic liver diseases

**FIGURE 5:**
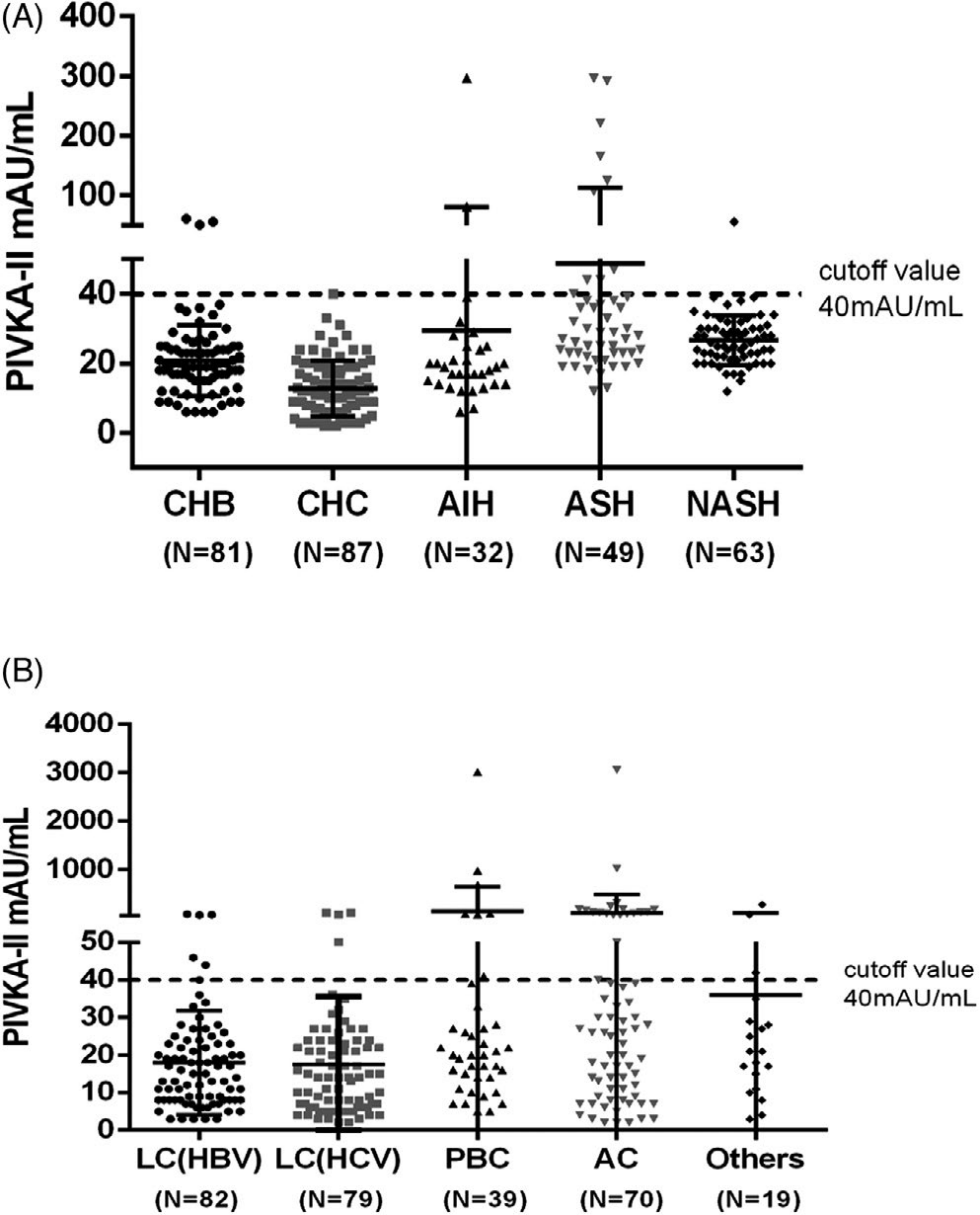
Distribution of PIVKA‐II in chronic liver diseases. Scatter plots of serum levels of PIVKA‐II in (A) the chronic hepatitis group and (B) the liver cirrhosis group. The chronic hepatitis group included chronic hepatitis B (CHB), chronic hepatitis C (CHC), alcoholic steatohepatitis (ASH), non‐alcoholic steatohepatitis (NASH), and autoimmune hepatitis (AIH) patients. The liver cirrhosis group included HBV‐related liver cirrhosis, HCV‐related liver cirrhosis, primary biliary cirrhosis (PBC), alcohol‐related cirrhosis (AC), and other cirrhosis patients. The number of patients in each group was indicated below, respectively

To better explore the specificity of PIVKA‐II, we observed the distribution of PIVKA‐II among chronic liver diseases with different etiologies in Figure 5. The mean value of PIVKA‐II in CH was 25.04 mAU/mL (range 2–297 mAU/mL), and the value of PIVKA‐II in chronic hepatitis B (mean 20.83 mAU/mL, range 6–61 mAU/mL) was close to it. Notably, the concentrations of PIVKA‐II in CHC were significantly lower (mean 12.83 mAU/mL, range 2–40 mAU/mL). Among CH, ASH had the highest concentration (mean 48.80 mAU/mL, range 12–296 mAU/mL). There is no doubt that the concentrations of PIVKA‐II in LC group (mean 56 mAU/mL, range 2–3057 mAU/mL) were comparatively higher than the CH group. AC also revealed the higher concentration (mean 101.7 mAU/mL, range 2–3057 mAU/mL) among the LC group. Above all, the positive rate of PIVKA‐II exceeding 40 mAU/mL cutoff value was 4.81% in 312 CH patients and 12.8% in 289 LC patients, respectively.

### 3.4 The optimal cutoff values of PIVKA‐II for diagnosing HCC in patients with CHB or cirrhosis

**FIGURE 6:**
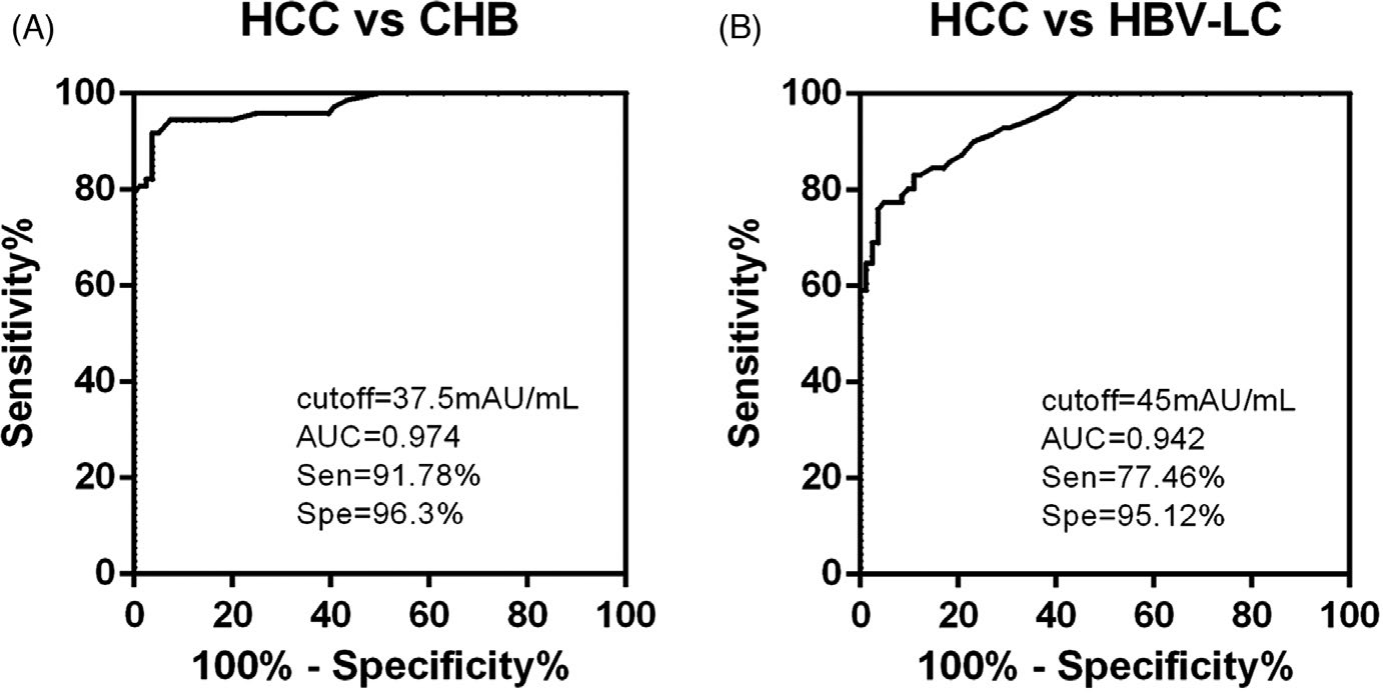
Optimal cutoff values of PIVKA‐II for diagnosing HBV‐related HCC. Receiver‐operating characteristic curves (ROC) and their corresponding area under the curve (AUC) values for (A) HBV‐related HCC patients without cirrhosis(*n* = 73) vs chronic hepatitis B (*n* = 81), (B) HBV‐related HCC with cirrhosis (*n* = 71) vs HBV‐related liver cirrhosis (*n* = 82)

Since the key determinant of HCC in China is HBV infection, HCC surveillance in patients with these contexts was significantly important. Therefore, we inquired the optimal cutoff values of PIVKA‐II in patients with CHB‐ or HBV‐related cirrhosis (Figure 6). PIVKA‐II at a cutoff value of 37.5 mAU/mL yielded an AUC of 0.9737, with a sensitivity of 91.78% and a specificity of 96.3%, in discriminating HBV‐related HCC patients without cirrhosis from CHB. PIVKA‐II at a cutoff value of 45 mAU/mL yielded an AUC area of 0.9419, with a sensitivity of 77.46% and a specificity of 95.12%, in discriminating HBV‐related HCC with cirrhosis from HBV‐related cirrhosis patients.

## 4 DISCUSSION

As the most widely used HCC marker, AFP always has a false‐negative rate from 30% to 40%,^26^ and its concentrations may be elevated in several nonspecific conditions other than HCC. After AFP, many studies have revealed that elevated PIVKA‐II is associated with tumor size and vascular invasion^27^ and has highly sensitivity and specificity for the diagnosis and prognosis monitoring of HCC, especially in HBV‐related liver disease.^28^ In this study, we investigated the diagnostic efficacy of PIVKA‐II in patients with different etiologies of chronic hepatitis and cirrhosis. The results showed that PIVKA‐II had a sensitivity of 84.08% and a specificity of 94.55% when distinguishing HCC from patients with different etiology of chronic hepatitis; a sensitivity of 84.08% and a specificity of 86.51% when distinguishing HCC from cirrhosis. In accordance with previous reports, these data indicated the excellent diagnostic efficacy of PIVKA‐II in liver cancer. Considering that AFP and PIVKA‐II are not correlated,^29^ the combination of AFP and PIVKA‐II measurement will have better diagnostic performances on HCC. Our data also validated that the combination of AFP and PIVKA‐II increases the detection rate of HCC.

The recent global burden of primary liver cancer has indicated that HBV was the leading cause of liver cancer occurrence and death, followed by HCV and alcohol.^5^ CHB patients without antiviral treatment may progress to severe fibrosis and cirrhosis at a rate of 2%–10% and the incidence of HCC in patients with cirrhosis is 3%−6%.^30^, ^31^ On the other hand, in non‐cirrhotic CHB patients, there is still 0.5%–1.0% incidence of HCC.^32^ A multi‐center investigation conducted by 112 hospitals in China enrolled 18,275 HCC patients and demonstrated that 80.50% cases had HBV infection and 80.12% cases had liver cirrhosis.^33^ All these data indicate the importance of HCC surveillance in patients with the context of CHB and cirrhosis in China. In this study, we inquired the optimal cutoff value of PIVKA‐II for diagnosing HCC in patients with CHB or cirrhosis. PIVKA‐II yielded a ROC curve area of 0.9737, with a sensitivity of 91.78% and a specificity of 96.3%, in discriminating HCC from CHB patients, at a cutoff of 37.5 mAU/mL. PIVKA‐II yielded a ROC curve area of 0.9419, with a sensitivity of 77.46% and a specificity of 95.12%, in discriminating HCC from HBV‐related cirrhosis patients, at a cutoff at 45 mAU/mL. These values were close to the cutoff value of 40 mAU/mL in the previous reports.^34^ Consistent with other reports, a specificity around 95.12%–96.3% revealed the superior discriminating power of PIVKA‐II under the context of CHB and cirrhosis than AFP, supporting the opinion that PIVKA‐II is more specific for HCC surveillance in HBV‐related high‐risk patients.

Although PIVKA‐II is regarded as a highly specific HCC biomarker, it is disturbed by vitamin K deficiency or antagonist drugs such as warfarin according to its characteristic as a prothrombin induced by vitamin K absence.^35^ Other research demonstrated that alcoholic liver disease and antibiotics usage could also influence PIVKA‐II.^36^ To address this issue, we investigated the distribution of PIVKA‐II among chronic liver diseases of different etiologies in this study. Consistent with other literature, alcohol‐related liver disease had higher PIVKA‐II concentrations regardless of cirrhosis context (ASH vs CHS: 48.80 vs 25.04; AC vs LC: 101.70 vs 55.99). Therefore, high PIVKA‐II concentrations in patients with ASH should be interpreted with careful deliberation. In addition, PIVKA‐II values in the patients with cirrhosis were comparatively higher than those in the patients with hepatitis (CH vs LC: 25.04 vs 55.99). Elevated PIVKA‐II may reflect the progressive liver damage by cirrhosis, and on the other hand, it may be due to the interferences such as obstructive jaundice and cholestasis.^37^ Although the above‐mentioned influencing factors were included, 4.81% of 312 patients with chronic hepatitis exceeded PIVKA‐II cutoff value (40 mAU/mL), and 12.8% of 289 patients with liver cirrhosis, revealing the satisfactory specificity of PIVKA‐II in chronic liver disease.

However, there are still some limitations in this research. First, we concentrated on HCC surveillance in HBV‐related live diseases, and thus, we mainly focused on HBV‐related patients. Due to the incidence of alcoholic and non‐alcoholic fatty liver diseases increases year by year, monitoring of liver cancer in these patients is needed in further research. Second, according to the recommendations of the Clinical and Laboratory Standards Institute (CLSI) guidelines for establishing reference intervals (EP28‐A3c), 120 observations are the minimum requirements for statistical significance for both parametric and non‐parametric data. Due to the time limitation, we did not gather the enough data especially in autoimmune hepatitis and steatohepatitis. Therefore, we investigated the tendency of PIVKA‐II distribution among chronic liver disease without statistical analysis.

## 5 CONCLUSION

In conclusion, our data indicated that PIVKA‐II had satisfactory diagnostic performances in patients with liver diseases of various etiologies and demonstrated that PIVKA‐II can be used as a screening or surveillance biomarker in HCC high‐risk populations. Furthermore, this research revealed that PIVKA‐II was less affected by the patients with HBV‐related liver disease, showing its superior diagnostic efficacy than AFP. We believe that the combination of AFP and PIVKA‐II will improve the diagnosis and the recurrence detection rate of HCC patients, increase the proportion of early treatment, ultimately improve the survival rate and prolong the life of HCC patients.
